# Phase-wise comparison of depression and stigma among tuberculosis patients undergoing treatment in Dhaka, Bangladesh

**DOI:** 10.1016/j.ijregi.2025.100790

**Published:** 2025-10-18

**Authors:** Dilkhush Jahan, Md Abdullah Saeed Khan, Sharmim Akter, Md. Sazid Rezwan, Israt Zahan Sarna, Md. Rahul Parvez, Golam Dastageer Prince, Salah Uddin Ahmed, Meerjady Sabrina Flora

**Affiliations:** 1 National Institute of Preventive and Social Medicine, Mohakhali, Dhaka, Bangladesh; 2 Directorate General of Health Services, Mohakhali, Dhaka, Bangladesh; 3 Mymensingh Medical College Hospital, Mymensingh, Bangladesh; 4 Rajshahi Medical College, Rajshahi, Bangladesh

**Keywords:** Tuberculosis, Depression, Stigma, Treatment phase, Bangladesh

## Abstract

**Objectives:**

Tuberculosis (TB) remains a significant global health concern with established links to depression and stigma. Both outcomes have been found to vary between treatment phases of TB. This study compared depression and stigma among patients with TB in the intensive and continuation treatment phases in Dhaka, Bangladesh.

**Methods:**

A cross-sectional comparative study was conducted during August and September 2023 among 111 patients in the intensive phase and 113 in the continuation phase of TB treatment (a total of 224) at directly observed treatment short-course centers. Depression was assessed using the Patient Health Questionnaire-9, and stigma was measured using Van Rie’s TB/HIV Stigma Scale. Multivariable logistic regression identified associated factors.

**Results:**

The prevalence of depression was 56.2% overall, with no significant difference between the intensive (59.5%) and continuation phases (53.1%). Anticipated stigma was more prevalent (74.6%) than social stigma (37.5%), with neither showing significant phase variation. Pulmonary TB was associated with both social stigma (odds ratio [OR] = 3.27, 95% confidence interval [CI]: 1.69-6.52) and anticipated stigma (OR = 3.29, 95% CI: 1.70-6.59). Living with family increased the odds of experiencing anticipated stigma, while patient counseling demonstrated protective effects. Depression was associated with treatment adherence difficulties (OR = 3.55, 95% CI: 1.75-7.51), persistent TB symptoms (OR = 2.23, 95% CI: 1.04-4.91), and social stigma (OR = 2.06, 95% CI: 1.02-4.22).

**Conclusions:**

The high prevalence of depression and persistent stigma throughout treatment highlight the need for continuous mental health support across all phases. TB care should integrate depression screening, stigma reduction strategies, and enhanced counseling to improve outcomes.

## Highlights

• Depression (56.2%), self-stigma (74.6%), and social stigma (37.5%): no phase difference.
• Pulmonary tuberculosis (TB) had three times higher odds of social and self-stigma compared with extrapulmonary TB.
• Living with family increased stigma risk, while counseling was protective.
• Depression among patients with TB is linked to treatment difficulty, symptoms, and stigma.

## Introduction

Tuberculosis (TB) continues to be a serious global health concern, particularly in high-burden countries like Bangladesh. The country ranks seventh among the world’s 30 highest TB-burden countries [1]. Caused by *Mycobacterium tuberculosis*, TB primarily affects the lungs but can involve almost any organ system. Globally, TB is the thirteenth leading cause of death and the leading infectious killer, surpassing HIV/AIDS [2]. In 2023, an estimated 10.8 million people developed TB [3], marking an approximate 1.9% increase from the previous year. The World Health Organization’s End TB Strategy aims to achieve zero deaths, disease, and suffering due to TB by 2035 [4]. Bangladesh’s National Tuberculosis Control Program has incorporated the directly observed treatment short-course (DOTS) strategy within its public health framework to combat this growing challenge. Despite being curable with appropriate treatment, TB remains stigmatized, with affected individuals often subjected to discrimination and social exclusion [5], leaving them vulnerable to mental health problems.

Stigma has been a longstanding barrier to the global control of TB. It can foster fear of discrimination and social isolation, often forcing individuals with TB to conceal their diagnosis, avoid seeking timely care, or discontinue treatment, leading to poor health outcomes and ongoing transmission in the community [5]. TB-related stigma arises from misconceptions about the contagious nature of the disease, its association with poverty, and the visible physical symptoms that accompany it. Studies have shown that stigma among patients with TB is highly prevalent [6], particularly in low- and middle-income countries, where it contributes to psychological distress, social isolation, and reduced quality of life [7]. In Bangladesh, where TB remains a major public health concern, stigma continues to hinder efforts toward early diagnosis, treatment adherence, and social integration [8]. Stigma associated with TB may occur in four forms [9]: anticipated stigma (fear of stigma), perceived community stigma (social stigma), enacted stigma (actual discrimination), and internalized stigma (self-stigmatization). Anticipated stigma may cause patients to hide their TB status, creating barriers to proper management. The negative social perception toward patients with TB not only exacerbates mental health issues such as depression but also diminishes treatment outcomes and increases the risk of disease complications and mortality [[10], [11], [12]]. When stigma is enacted in the community, patients experience various forms of abuse and bereavement. Self-stigma may cause individuals to devalue themselves and their lives, often leading to a diminished sense of meaning. Patients with TB who struggle with self-stigma are more likely to develop depression, negatively affecting their compliance [9].

The interaction between TB and mental health, particularly depression, can create a vicious cycle. The prolonged treatment duration, physical symptoms such as fatigue, cough, and weight loss, alongside fears of contagion and social stigma, elevate the risk of depression among patients with TB [12]. Additionally, the presence of depression can impair immune function, increase stress, and reduce self-care behavior, resulting in increased susceptibility to TB infection. Studies in different countries have shown high rates of depression among patients with TB— 56% in Pakistan [10], 31.1% in Ethiopia [13], and 48.6% in Nigeria [14], with a pooled prevalence of 45.19% around the world [15]. In Bangladesh, a depression prevalence of 39.8% was detected among patients with TB a decade ago in a tertiary care hospital [16]. Among patients with multidrug-resistant (MDR) TB, the prevalence was found to be 33.8% at a specialized chest hospital [17].

The prevalence and severity of depression among patients with TB have been documented to vary between the intensive and continuation phases of treatment [[18], [19], [20]], with the former phase patients being more likely to have depression [19,21,22]. During the intensive phase, patients often experience more pronounced physical symptoms, frequent clinic visits, a higher pill burden, and side effects of anti-TB medications, which could heighten their emotional distress and vulnerability to depression. The continuation phase, while clinically less demanding, may still carry significant psychological burdens due to prolonged social isolation, treatment fatigue, and ongoing stigma, especially if symptoms persist or if patients face challenges reintegrating into daily life. Similarly, stigma has been reported to vary across treatment phases, with some studies reporting a reduction [23,24] and others reporting persistence of stigma [25] between the intensive and continuation phases. Social stigma during the early treatment phase may stem from fears of contagion and misconceptions about TB [8], whereas self-stigma in the later phase [24] may be sustained by internalized shame, guilt, or community rejection. These differences have important implications for decisions on mental health support and health education interventions required for the successful management of TB.

Existing studies have largely focused on the overall magnitude of depression and stigma among patients with TB. Moreover, the stigma faced by patients with TB has been less explored in Bangladesh. Therefore, this study aimed to compare the prevalence of depression and stigma among patients with TB in the intensive and continuation phases of treatment in Bangladesh, addressing a critical gap in the current literature.

## Materials and methods

### Study place, participants, and duration

A cross-sectional comparative study was carried out from August to September 2023 at DOTS centers of non-governmental organizations in Dhaka, Bangladesh. The study population consisted of pulmonary and extrapulmonary patients with TB aged ≥18 years who had undergone uninterrupted anti-TB treatment for the last 15 days. Pregnant or breastfeeding women (up to 3 months postpartum) and patients with a prior history of mental disorders were excluded to avoid confounding effects.

### Sample size and sampling

The sample size was calculated based on depression prevalence rates among patients with TB in the intensive phase (P_1_ = 67.8%) and continuation phase (P_2_ = 41.3%) of treatment from a previous study [18]. Assuming a 95% confidence level (Z_α_ = 1.96) and 80% power (Z_β_ = 0.84), the sample size was calculated using the following formula:

$$n=\frac{P_{1}\left(100-P_{1}\right)+P_{2}\left(100-P_{2}\right)}{{\left(P_{1}-P_{2}\right)}^{2}}\times {\left(Z_{\alpha }+Z_{\beta }\right)}^{2}$$

Plugging in those numbers in the equation gave a sample size of 51.43 per group. Adding 10% non-response, it yielded 51.43 + 10% of 51.43 = 56.58. To increase the reliability and power of the study [26], we doubled the sample to get the final estimate of 113.16 ∼ 113 for each group. However, a total of 111 patients in the intensive phase and 113 patients in the continuation phase were selected from the study sites during the study period.

### Data collection technique

Convenience sampling was used to select participants from DOTS centers. Data was collected via face-to-face interviews using a semi-structured questionnaire administered on Android tablets via Google Forms. Before participation, respondents were informed about the study objectives, and informed consent was obtained. Privacy and confidentiality were strictly maintained.

### Data collection instruments

A semi-structured questionnaire was used, incorporating sociodemographic characteristics (e.g., age, sex, education, occupation, marital status, being an earning member, and living status with family); information related to TB (TB type, TB symptoms at presentation, persistence of symptoms after initiation of treatment, treatment phase, regularity of taking anti-TB treatment [ATT], reasons for missing any doses, difficulty taking ATT daily, side effects experienced or recorded by the physician during the treatment, presence of chronic diseases, presence of HIV, ever counseled by the DOTS personnel regarding TB treatment); information related to TB stigma assessed using Van Rie’s TB/HIV Stigma Scale [27]; and information related to depressive symptoms assessed using the Patient Health Questionnaire-9 (PHQ-9) [28].

### Van Rie’s stigma scale

The TB stigma part of the scale was taken. This 23-item scale evaluates stigma from two perspectives. One, the community perspective on stigma (11 items) measures perceived discrimination, an indirect measure of community-perceived stigma, or, in other words, social stigma. Two, the patient perspective on stigma (12 items) assesses anticipated stigma. All the statements have four responses: strongly disagree (score: 0), disagree (1), agree (2), and strongly agree (3). The total score is calculated after adding the item-specific scores, which range between 0 and 33 for the community perspective (social stigma) and between 0 and 36 for the patient's perspective (anticipated stigma). A total score of 0 was considered as having an absence of stigma, and a score of ≥ 1 indicated the presence of stigma. A Bangla-translated version of the TB stigma part of Van Rie’s scale was collected from an unpublished thesis exploring stigma among patients with TB. The translation was done by an expert English user and was reviewed by the investigator team. The instrument was then back-translated by another expert English user to match the original version. Then, the two versions (English and Bangla) were again reviewed by the investigators. In this study, we first pretested the Bangla version among a sample of 20 patients with TB and found the items to be compatible with their comprehension and understanding. Besides, the social stigma and anticipated stigma parts of the scale showed excellent (Cronbach’s α = 0.96) and good (α = 0.88) internal consistency (reliability), respectively, among our samples.

### Patient health questionnaire-9

The PHQ-9 scale was validated for use among adults in Bangladesh [29]. We used the validated Bangla version of the instrument in this study. It screens depression severity via nine items aligned with DSM-5 criteria. Each item is scored 0-3 (0 = not at all, 1 = several days, 2 = more than half the days, and 3 = nearly every day), yielding a total score of 0-27. A score of ≤ 4 was considered as absence of depression, and a score >4 was considered as presence of depression in our study. The scale showed excellent reliability (α = 0.90) among our participants.

### Data analysis

Data recorded in Google Sheets was downloaded, cleaned, and saved as a comma-separated value file for analysis using R Studio Version 2024.12.1. Normality of the continuous data (age and monthly income) was assessed using a histogram with a normal curve and the Shapiro–Wilk test. The descriptive statistics were expressed as n (%) for categorical variables, mean (Standard deviation [SD]) for age, and median (interquartile range [IQR]) for monthly family income. Pearson’s chi-square test and Fisher’s exact test were used to assess associations between categorical variables. Mean age was compared using Welch’s two-sample *t*-test. Odds ratios (ORs) with 95% confidence intervals (CIs) were calculated for risk factors through multivariable logistic regression analysis. All the sociodemographic and TB-related characteristics were considered for regression except those with inapplicability across all samples (regularity of income and reasons for feeling difficulty taking ATT) and very low frequency (family history of mental health).

### Ethical considerations

Ethical approval was obtained from the National Institute of Preventive and Social Medicine (NIPSOM) (Memo no: NIPSOM/IRB/2023/06). Participants provided informed consent before inclusion. All procedures were conducted following the guidelines laid down in the Declaration of Helsinki.

## Results

The mean (SD) age of the participants was 40.68 (15.99) years. Males comprised 61.16% overall, with 54.95% in the intensive phase and 67.26% in the continuation phase. Most participants (45.54%) had education up to secondary school certificate, while 21.88% had no formal education. The majority were married (77.23%) and living with family (96.41%). Occupationally, housewives (24.55%), job holders (16.07%), and unemployed individuals (15.17%) were most common, with unemployment higher in the intensive (18.12%) than in the continuation phase (9.73%). More than half (55.80%) were earning members, with similar proportions across phases, though income regularity was higher in the intensive phase (74.60% vs 64.62%). The median (IQR) monthly family income was 30,000 Bangladeshi Taka (20,000-50,000) overall, slightly higher in the intensive phase. Noncommunicable diseases were present in 27.68%, primarily diabetes (22.32%) and hypertension (17.41%). Of all, 57.59% had pulmonary TB and 42.41% extrapulmonary TB, with pulmonary TB more frequent in the continuation phase (65.49%) and extrapulmonary in the intensive phase (49.55%). All patients except three were on daily ATT (98.66%), with 33.04% reporting difficulty, more common in the intensive phase (43.34%). Persistence of TB symptoms was reported by 25.45%, predominantly in the intensive phase (37.84%). Side effects of ATT were common (67.41%), especially itching (55.77%), anorexia (43.31%), and nausea (41.18%), mostly reported in the intensive phase. Counseling was received by 93.75% of patients and 62.50% of families, with slightly higher rates reported by those in the intensive phase (Table 1).

**Table 1:** Sociodemographic, tuberculosis, and chronic disease-related characteristics of the respondents by treatment phase.

		Treatment phase	
Characteristic	Overall (N = 224) n (%)	Intensive phase (N = 111) n (%)	Continuation phase (N = 113) n (%)
**Age (years), mean (SD)**	40.68 (15.99)	40.60 (16.12)	40.76 (15.94)
**Sex**			
Male	137 (61.16)	61 (54.95)	76 (67.26)
Female	87 (38.84)	50 (45.05)	37 (32.74)
**Educational qualification**			
No formal education	49 (21.88)	20 (18.02)	29 (25.66)
Up to SSC	102 (45.54)	51 (45.95)	51 (45.13)
Up to HSC	26 (11.61)	15 (13.51)	11 (9.73)
Graduation	47 (20.98)	25 (22.52)	22 (19.47)
**Marital status**			
Married	173 (77.23)	80 (72.07)	93 (82.30)
Unmarried	33 (14.73)	18 (16.22)	15 (13.27)
Widow	12 (5.36)	7 (6.31)	5 (4.42)
Divorced	5 (2.23)	5 (4.50)	0 (0.00)
Separated	1 (0.45)	1 (0.90)	0 (0.00)
**Living status**			
Living with family	215 (96.41)	107 (96.40)	108 (96.43)
Living alone	8 (3.59)	4 (3.60)	4 (3.57)
**Occupation**			
Housewife	55 (24.55)	26 (23.42)	29 (25.66)
Job holder	36 (16.07)	17 (15.32)	19 (16.81)
Business	29 (12.95)	12 (10.81)	17 (15.04)
Retired	17 (7.59)	9 (8.11)	8 (7.08)
Student	17 (7.59)	9 (8.11)	8 (7.08)
Driver	12 (5.36)	8 (7.21)	4 (3.54)
Fired from job	10 (4.46)	7 (6.31)	3 (2.65)
Day laborer	7 (3.13)	1 (0.90)	6 (5.31)
Rickshaw puller	7 (3.13)	3 (2.70)	4 (3.54)
Maid servant	2 (0.89)	2 (1.80)	0 (0.00)
Others	8 (3.57)	4 (3.60)	4 (3.54)
Unemployed	34 (15.17)	20 (18.12)	14 (9.73)
**Is the participant earning person of the family**			
Yes	125 (55.80)	62 (55.86)	63 (55.75)
No	99 (44.20)	49 (44.14)	50 (44.25)
**Regularity of income**			
Regular	89 (69.53)	47 (74.60)	42 (64.62)
Irregular	39 (30.47)	16 (25.40)	23 (35.38)
**Monthly family income (BDT), median (interquartile range)**	30,000 (20,000, 50,000)	30,000 (20,000, 60,000)	30,000 (20,000, 50,000)
**Any noncommunicable disease**	62 (27.68)	30 (27.68)	32 (28.32)
Diabetes Mellitus	50 (22.32)	21 (18.92)	29 (25.66)
Hypertension	39 (17.41)	21 (18.92)	18 (15.93)
**Site of TB**			
Pulmonary TB	129 (57.59)	55 (49.55)	74 (65.49)
Extra Pulmonary TB	95 (42.41)	56 (50.45)	39 (34.51)
**Takes ATT daily**	221 (98.66)	110 (99.10)	111 (98.23)
**Reasons for missed ATTs**			
Feeling better	1 (33.33)	0 (0.00)	1 (33.33)
Others	1 (33.33)	0 (0.00)	1 (33.33)
Unknown	1 (33.33)	0 (0.00)	1 (33.33)
**Feels difficulty in taking ATT daily**	74 (33.04)	47 (43.34)	27 (23.89)
**Persistence of TB symptoms after treatment initiation**	57 (25.45)	42 (37.84)	15 (13.27)
**Any side effects of ATT developed**	151 (67.41)	96 (86.49)	55 (48.67))
Itching	87 (55.77)	65 (65.66)	22 (38.60)
Anorexia	69 (43.31)	44 (44.44)	24 (41.38)
Nausea	63 (41.18)	42 (42.86)	21 (38.18)
Visual impairment	28 (34.15)	8 (18.60)	20 (51.28)
Vomiting	35 (22.44)	23 (23.23)	12 (21.05)
Joint pain	36 (23.08)	22 (22.22)	14 (24.56)
Neuropathy	23 (14.84)	7 (7.14)	16 (28.07)
Skin rash	14 (9.09)	11 (11.22)	3 (5.36)
Vertigo	14 (8.97)	9 (9.09)	5 (8.77)
**Counselling received by the patient**	210 (93.75)	107 (96.40)	103 (91.15)
**Counselling received by the family**	140 (62.50)	77 (69.37)	63 (55.75)

ATT, anti-TB treatment; BDT, Bangladeshi Taka; HSC, higher secondary certificate; SSC, secondary school certificate; TB, tuberculosis.

**Figure 1:**
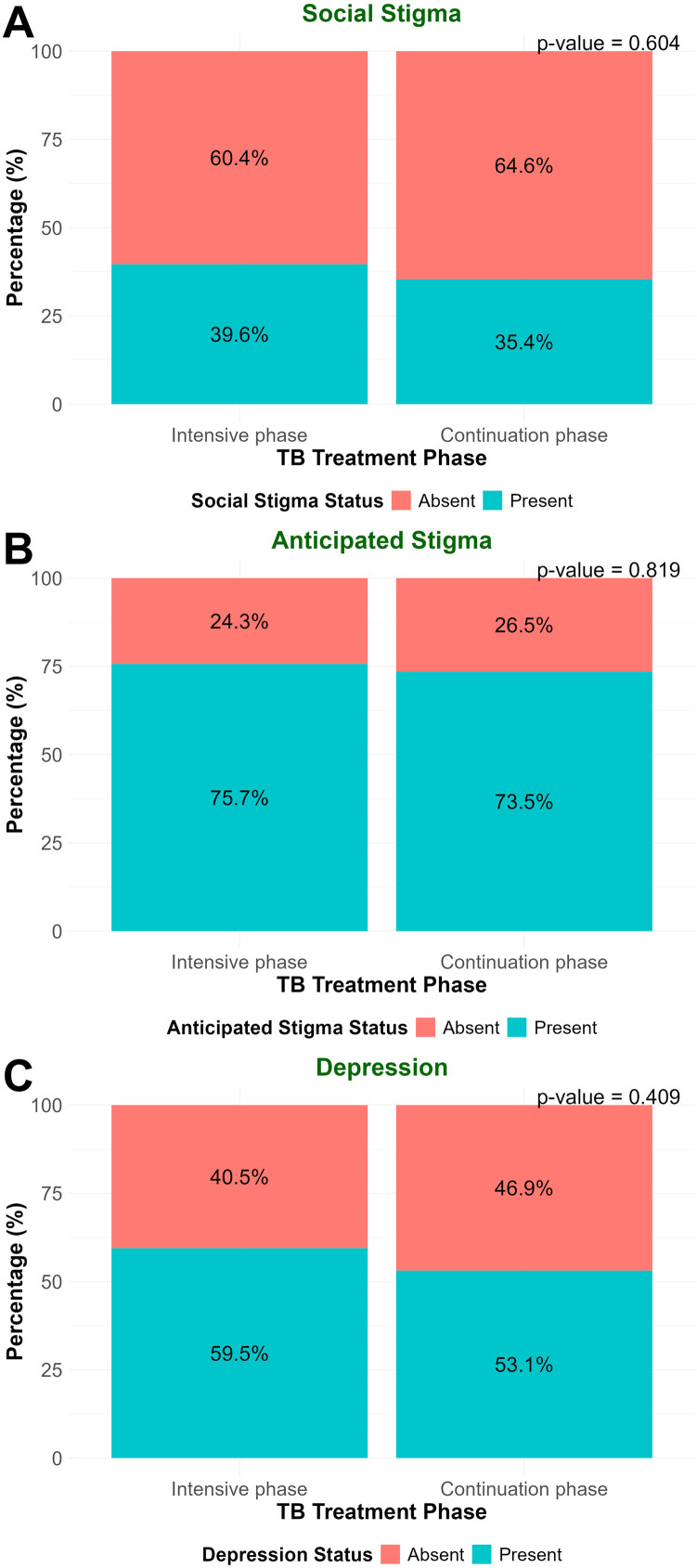
Comparison of social stigma, anticipated stigma, and depression among TB patients between intensive and continuation phases of treatment

The proportions of social stigma, anticipated stigma, and depression were 39.6%, 75.7%, and 59.5%, respectively, among patients in the intensive phase, and 35.4%, 73.5%, and 53.1% among patients in the continuation phase. The differences were not statistically significant (*P* = 0.604, *P* = 0.819 and *P* = 0.409, respectively) (Figure 1). Of all participants, 37.5% had social stigma, 74.6% had anticipated stigma, and 56.2% had depression (Figure S1). Distribution of responses to individual items of Van Rie’s stigma scale and PHQ-9 scale is described in Supplementary Tables S1-S3.

Younger age (*P* = 0.033) and having pulmonary TB were significantly associated with higher social stigma. Patients with pulmonary TB had 3.27 times higher odds of experiencing social stigma (95% CI: 1.69-6.52, *P* <0.001). Living with family was also associated with higher odds of social stigma (OR = 10.43, 95% CI: 1.53-211.67, *P* = 0.041). In contrast, receiving patient counseling reduced the odds (OR = 0.22, 95% CI: 0.06-0.82, *P* = 0.027). The phase of treatment was not associated with social stigma (OR = 0.73, 95% CI: 0.37-1.43, *P* = 0.354) (Table 2).

**Table 2:** Characteristics of participants by social stigma.

	Social stigma			Multivariable logistic regression		
Characteristic	Absent (N = 140)	Present (N = 84)	*P*-value	Odds ratio	95% confidence interval	p-value
**TB treatment phase, n (%)**			0.512^a^			
Intensive phase	67 (60.36)	44 (39.64)		—	—	
Continuation phase	73 (64.60)	40 (35.40)		0.73	0.37-1.43	0.354
**Age (years), mean (SD)**	42.43 (16.15)	37.77 (15.39)	**0.033**^b^	0.97	0.94-1.00	0.053
**Sex, n (%)**			0.860^a^			
Female	55 (63.22)	32 (36.78)		—	—	
Male	85 (62.04)	52 (37.96)		1.39	0.64-3.07	0.408
**Educational status, n (%)**			0.900^a^			
Formal education	109 (62.29)	66 (37.71)		—	—	
No formal education	31 (63.27)	18 (36.73)		1.46	0.58-3.69	0.425
**Marital status, n (%)**			0.109^a^			
Married	113 (65.32)	60 (34.68)		—	—	
Single	27 (52.94)	24 (47.06)		1.66	0.80-3.48	0.174
**Living status, n (%)**			0.264^c^			
Living alone	7 (87.50)	1 (12.50)		—	—	
Living with family	132 (61.40)	83 (38.60)		10.43	1.53-211.67	**0.041**
**Occupation, n (%)**			0.972^a^			
Employed	63 (62.38)	38 (37.62)		—	—	
Unemployed	77 (62.60)	46 (37.40)		0.90	0.34-2.36	0.837
**Participant is the earning member of the family, n (%)**			0.972^a^			
No	62 (62.63)	37 (37.37)		—	—	
Yes	78 (62.40)	47 (37.60)		0.81	0.28-2.27	0.687
**Monthly family income (BDT), n (%)**			0.677^a^			
More than 30,000	61 (61.00)	39 (39.00)		—	—	
Up to 30,000	79 (63.71)	45 (36.29)		0.59	0.29-1.17	0.134
**Site of TB, n (%)**			**0.001**^a^			
Extra Pulmonary TB	71 (74.74)	24 (25.26)		—	—	
Pulmonary TB	69 (53.49)	60 (46.51)		3.27	1.69-6.52	**<0.001**
**Persistence of TB symptoms after treatment initiation, n (%)**			0.905^a^			
No	104 (62.28)	63 (37.72)		—	—	
Yes	36 (63.16)	21 (36.84)		1.09	0.52-2.28	0.811
**Any side effects of ATT developed, n (%)**			0.484^a^			
No	48 (65.75)	25 (34.25)		—	—	
Yes	92 (60.93)	59 (39.07)		1.36	0.67-2.80	0.404
**Any noncommunicable disease, n (%)**			0.316^a^			
Absent	98 (60.49)	64 (39.51)		—	—	
Present	42 (67.74)	20 (32.26)		1.22	0.48-3.13	0.674
**Counselling received by the patient, n (%)**			0.117^a^			
No	6 (42.86)	8 (57.14)		—	—	
Yes	134 (63.81)	76 (36.19)		0.22	0.06-0.82	**0.027**
**Counselling received by the family, n (%)**			0.669^a^			
No	51 (60.71)	33 (39.29)		—	—	
Yes	89 (63.57)	51 (36.43)		0.77	0.38-1.55	0.467

a Pearson’s chi-squared test.

b Welch two-sample *t*-test.

c Fisher's exact test. ATT, anti-TB treatment; BDT, Bangladeshi Taka; TB, tuberculosis.

For anticipated stigma, patients with pulmonary TB had 3.29 times higher odds (95% CI: 1.70-6.59, *P* <0.001). Living with family (OR = 9.91, 95% CI: 1.48-199.64, *P* = 0.044) increased anticipated stigma, while patient counseling was protective (OR = 0.22, 95% CI: 0.06-0.83, *P* = 0.028). Other variables, including age, sex, education, and income, showed no significant associations. The phase of treatment was not associated with anticipated stigma (OR = 0.74, 95% CI: 0.37-1.47, *P* = 0.394) (Table 3).

**Table 3:** Characteristics of participants by anticipated stigma.

	Anticipated stigma			Multivariable logistic regression		
Characteristic	Absent (N = 57)	Present (N = 167)	*P*-value	Odds ratio	95% confidence interval	*P*-value
**TB treatment phase, n (%)**			0.702^a^			
Intensive phase	27 (24.32)	84 (75.68)		—	—	
Continuation phase	30 (26.55)	83 (73.45)		0.74	0.37-1.47	0.394
**Age (years), mean (SD)**	42.39 (16.09)	40.10 (15.96)	0.356^b^	0.97	0.94-1.00	**0.045**
**Sex, n (%)**			0.126^a^			
Female	27 (31.03)	60 (68.97)		—	—	
Male	30 (21.90)	107 (78.10)		1.45	0.66-3.22	0.359
**Educational status, n (%)**			0.570^a^			
Formal education	43 (24.57)	132 (75.43)		—	—	
No formal education	14 (28.57)	35 (71.43)		1.53	0.61-3.89	0.367
**Marital status, n (%)**			0.469^a^			
Married	46 (26.59)	127 (73.41)		—	—	
Single	11 (21.57)	40 (78.43)		1.61	0.77-3.37	0.207
**Living status, n (%)**			0.424^c^			
Living alone	3 (37.50)	5 (62.50)		—	—	
Living with family	54 (25.12)	161 (74.88)		9.91	1.48-199.64	**0.044**
**Occupation, n (%)**			0.829^a^			
Employed	25 (24.75)	76 (75.25)		—	—	
Unemployed	32 (26.02)	91 (73.98)		0.81	0.29-2.15	0.669
**Participant is the earning member of the family, n (%)**			0.577^a^			
No	27 (27.27)	72 (72.73)		—	—	
Yes	30 (24.00)	95 (76.00)		0.77	0.26-2.19	0.634
**Monthly family income (BDT), n (%)**			0.890^a^			
More than 30,000	25 (25.00)	75 (75.00)		—	—	
Up to 30,000	32 (25.81)	92 (74.19)		0.58	0.29-1.16	0.129
**Site of TB, n (%)**			**0.015**^a^			
Extra pulmonary TB	32 (33.68)	63 (66.32)		—	—	
Pulmonary TB	25 (19.38)	104 (80.62)		3.29	1.70-6.59	**<0.001**
**Persistence of TB symptoms after treatment initiation, n (%)**			0.379^a^			
No	40 (23.95)	127 (76.05)		—	—	
Yes	17 (29.82)	40 (70.18)		1.06	0.50-2.23	0.873
**Feels difficulty in taking ATT daily, n (%)**			0.479^a^			
No	36 (24.00)	114 (76.00)		—	—	
Yes	21 (28.38)	53 (71.62)		1.51	0.76-3.02	0.238
**Any side effects of ATT developed, n (%)**			0.890^a^			
No	19 (26.03)	54 (73.97)		—	—	
Yes	38 (25.17)	113 (74.83)		1.25	0.61-2.62	0.542
**Any noncommunicable disease, n (%)**			0.790^a^			
Absent	42 (25.93)	120 (74.07)		—	—	
Present	15 (24.19)	47 (75.81)		1.25	0.49-3.23	0.638
**Counseling received, n (%)**			0.527^c^			
No	2 (14.29)	12 (85.71)		—	—	
Yes	55 (26.19)	155 (73.81)		0.22	0.06-0.83	**0.028**
**Family was counseled, n (%)**			0.663^a^			
No	20 (23.81)	64 (76.19)		—	—	
Yes	37 (26.43)	103 (73.57)		0.78	0.39-1.57	0.482

a Pearson’s chi-squared test.

b Welch two-sample *t*-test.

c Fisher's exact test ATT, anti-TB treatment; BDT, Bangladeshi Taka; TB, tuberculosis.

Participants who had difficulty taking ATT daily had significantly higher odds of depression (OR = 3.55, 95% CI: 1.75-7.51, *P* <0.001). Persistence of TB symptoms after treatment initiation was also associated with increased depression (OR = 2.23, 95% CI: 1.04-4.91, *P* = 0.042). Additionally, those experiencing social stigma had a higher risk of depression (OR = 2.06, 95% CI: 1.02-4.22, *P* = 0.046). Unemployed participants tended to have higher odds of depression (OR = 2.40, 95% CI: 0.88-7.03, *P* = 0.096), although not statistically significant. No significant associations were found with other sociodemographic characteristics, TB type, comorbidities, or counseling. The phase of treatment was not associated with depression (OR = 1.29, 95% CI: 0.65-2.61, *P* = 0.470) (Table 4).

**Table 4:** Characteristics of participants by depression.

	Depression			Multivariable logistic regression		
Characteristic	Absent (N = 98) n (%)	Present (N = 126) n (%)	*P*-value	Odds ratio	95% confidence interval	*P*-value
**TB treatment phase, n (%)**			0.337^a^			
Continuation phase	53 (46.90)	60 (53.10)		1.29	0.65-2.61	0.470
Intensive phase	45 (40.54)	66 (59.46)		—	—	
**Age (years), mean (SD)**	41.78 (15.51)	39.83 (16.36)	0.365^b^	1.01	0.98-1.04	0.720
**Sex, n (%)**			0.094^a^			
Male	66 (48.18)	71 (51.82)		0.99	0.44-2.24	0.979
Female	32 (36.78)	55 (63.22)		—	—	
**Educational status, n (%)**			0.887^a^			
Formal education	77 (44.00)	98 (56.00)		—	—	
No formal education	21 (42.86)	28 (57.14)		0.89	0.36-2.23	0.805
**Marital status, n (%)**			**0.019**^a^			
Married	83 (47.98)	90 (52.02)		—	—	
Single	15 (29.41)	36 (70.59)		1.88	0.86-4.25	0.119
**Living status, n (%)**			>0.999^c^			
Living with family	95 (44.19)	120 (55.81)		0.98	0.17-4.88	0.976
Living alone	3 (37.50)	5 (62.50)		—	—	
**Occupation, n (%)**			**0.003**^a^			
Unemployed	43 (34.96)	80 (65.04)		2.40	0.88-7.03	0.096
Employed	55 (54.46)	46 (45.54)		—	—	
**Participant is the earning member of the family, n (%)**			**0.047**^a^			
Yes	62 (49.60)	63 (50.40)		1.30	0.44-4.01	0.641
No	36 (36.36)	63 (63.64)		—	—	
**Monthly family income (BDT), n (%)**			0.379^a^			
Up to 30,000	51 (41.13)	73 (58.87)		1.24	0.62- 2.50	0.542
More than 30,000	47 (47.00)	53 (53.00)		—	—	
**Site of TB, n (%)**			0.485^a^			
Pulmonary TB	59 (45.74)	70 (54.26)		0.91	0.46-1.81	0.790
Extra Pulmonary TB	39 (41.05)	56 (58.95)		—	—	
**Feels difficulty in taking ATT daily, n (%)**			**<0.001**^a^			
No	82 (54.67)	68 (45.33)		—	—	
Yes	16 (21.62)	58 (78.38)		3.55	1.75-7.51	**<0.001**
**Persistence of TB symptoms after treatment initiation, n (%)**			**0.014**^a^			
No	81 (48.50)	86 (51.50)		—	—	
Yes	17 (29.82)	40 (70.18)		2.23	1.04-4.91	**0.042**
**Any side effects of ATT developed, n (%)**			0.146^a^			
Yes	61 (40.40)	90 (59.60)		1.09	0.53-2.23	0.805
No	37 (50.68)	36 (49.32)		—	—	
**Any noncommunicable disease, n (%)**			0.387^a^			
Absent	68 (41.98)	94 (58.02)		—	—	
Present	30 (48.39)	32 (51.61)		0.73	0.29-1.81	0.499
**Counseling received, n (%)**			0.237^a^			
Yes	94 (44.76)	116 (55.24)		1.01	0.23-3.99	0.991
No	4 (28.57)	10 (71.43)		—	—	
**Family was counseled, n (%)**			0.186^a^			
Yes	66 (47.14)	74 (52.86)		0.58	0.28-1.17	0.132
No	32 (38.10)	52 (61.90)		—	—	
**Social stigma, n (%)**			**0.015**^a^			
Absent	70 (50.00)	70 (50.00)		—	—	
Present	28 (33.33)	56 (66.67)		2.06	1.02-4.22	**0.046**
**Anticipated stigma, n (%)**			0.524^2^			
Absent	27 (47.37)	30 (52.63)		—	—	
Present	71 (42.51)	96 (57.49)		1.00	0.48-2.12	0.991
**Family history of mental disorder, n (%)**			0.258^c^			
No	98 (44.34)	123 (55.66)				
Yes	0 (0.00)	3 (100.00)				

a Pearson’s chi-squared test.

b Welch two sample *t*-test.

c Fisher’s exact test. ATT, Anti-TB Treatment; BDT, Bangladeshi Taka; TB, tuberculosis.

## Discussion

This cross-sectional study compared the prevalence of depression and stigma among patients with TB in the intensive and continuation phases of treatment in Dhaka, Bangladesh. Our findings revealed high rates of depression (56.2%), anticipated stigma (74.6%), and social stigma (37.5%) among patients with TB overall, with no statistically significant differences between treatment phases. These results contrast with some previous studies showing phase-dependent variations in psychological burden among patients with TB.

The prevalence of depression among our study participants (56.2%) is higher than previously reported in Bangladesh [16,17] (39.8% at a tertiary care hospital and 33.8% among MDR-patients with TB), indicating that depression among patients with TB may be more prevalent than previously recognized. This finding aligns with the global pooled prevalence of 45.19% reported by Duko et al. [15] and is comparable to rates reported in Pakistan (56%) [10], though higher than those in Ethiopia (31.1%) [13] and Nigeria (48.6%) [14]. The high depression prevalence in our study underscores the significant mental health burden associated with TB in Bangladesh.

Contrary to our initial hypothesis and some previous studies, we found no significant difference in depression prevalence between the intensive and continuation phases. This differs from findings by Abdurahman et al. [18], Amha et al. [19], Kamble et al. [22], Rouf et al. [20], and Vaidya et al. [21], who reported higher depression rates during the intensive phase. Our contradictory finding might be explained by the continued psychological burden throughout the treatment period, possibly related to persistent stigma, ongoing social isolation, or economic hardships that extend beyond the intensive phase.

The high prevalence of anticipated stigma (74.6%) compared to social stigma (37.5%) was concordant with findings from other studies [25]. This suggests that the fear of stigma and associated feelings of shame and guilt may be more pervasive than actual discriminatory attitudes from the community. The significant proportion of patients experiencing anticipated stigma in both treatment phases suggests that feelings of shame and social devaluation persist throughout treatment, highlighting the need for continuous psychological support beyond the intensive phase among our patients. Our findings regarding stigma across treatment phases contrast with some previous research. Though Mohammedhussein et al. [23] and Duko et al. [15] reported a reduction in perceived stigma between the intensive and continuation phases, our study, similar to Dixit et al. [25], found no significant difference. This persistent anticipated stigma throughout treatment might be attributed to the deeply rooted misconceptions about TB in Bangladeshi society, as previously described by Dutta et al. [8]. They noted that patients with TB are often negatively stereotyped as a different, less powerful, and separated group of people in society because of prevalent social perceptions and prejudices against the disease [8]. Prejudices that the disease is a curse from God or a misfortune resulting from sin often stem from false religious or traditional beliefs, which require years of education and training to reshape and are often impossible to eliminate entirely. Hence, to improve people's stance toward patients with TB, continuous education and long-term, collaborative awareness-building efforts involving religious and community leaders might be needed.

Our multivariable analysis revealed several important associations with stigma and depression. Pulmonary TB was significantly associated with both social and anticipated stigma, with affected patients having over three times higher odds of experiencing stigma compared to those with extrapulmonary TB. This association likely stems from the perceived infectiousness of pulmonary TB, leading to greater fear of transmission and subsequent discrimination. Similar findings have been reported in previous studies, highlighting how the visible respiratory symptoms and concerns about contagion often drive TB-related stigma [5,30].

Living with family emerged as a significant predictor of both social and anticipated stigma, with affected individuals having approximately 10 times higher odds of experiencing stigma. This finding might reflect heightened awareness of negative attitudes within close family circles or the internalization of family members’ fears and misconceptions. The proximity of family members might also intensify feelings of guilt regarding potential disease transmission. This finding is particularly concerning given that family support is traditionally considered a protective factor against psychological distress. Nevertheless, Kılıç et al. [31] have shown that living with family can have both positive and negative influences on the perceived stigma of patients with TB. Parents of children with TB often refrain from starting treatment because of the fear of stigma, loss of custody, or partners leaving the family.

In contrast, patient counseling demonstrated a protective effect against both social and anticipated stigma, reducing the odds by approximately 78%. Counseling was demonstrated to reduce treatment defaulting by 13% in a randomized controlled trial in Pakistan [32], endorsing our finding. This also highlights the critical role of psychoeducation and counseling in challenging misconceptions, addressing fears, and mitigating stigma. It supports the integration of mental health support within TB treatment programs. Although our questionnaire did not include queries regarding the specifics of counseling, while explaining the question, we found that counseling in our setting mostly involved informing about the importance of adherence to treatment, the curability of TB, side effects of drugs, when to stop treatment if side effects occur and consult doctors, how and when TB spreads, and providing psychological and emotional support when needed. Both patients and their attendants were targeted for better compliance after counseling. However, a structured counseling pattern with additional support on demand would be the best way to prevent patients with TB from falling into depression and/or stigma.

For depression, difficulty taking ATT daily was the strongest predictor, with affected patients having 3.55 times higher odds of experiencing depression. This association may be bidirectional—medication side effects and regimen complexity may contribute to depression, while depression can impair treatment adherence [33]. The persistence of TB symptoms after treatment initiation also increased depression risk, likely due to prolonged physical discomfort and associated functional limitations.

Importantly, social stigma was significantly associated with depression, with affected patients having twice the odds of experiencing depressive symptoms. This finding aligns with the established relationship between stigma and depression, emphasizing how negative social attitudes can exacerbate mental health challenges among patients with TB [33]. While not statistically significant in our analysis, unemployment showed a trend toward association with depression, suggesting that economic factors might influence psychological well-being among patients with TB.

Our study has several strengths including the high reliability statistics of the assessment tools, a comprehensive analysis of both depression and stigma within the same population, and the comparison between treatment phases. However, certain limitations warrant consideration. The cross-sectional design precludes causal inferences and the ability to track changes in individual patients over time. The convenience sampling approach may limit generalizability, and the use of self-reported measures could not preclude the subjectivity of responses. In other words, there remained a risk of social desirability bias. Our study did not assess the self-stigma and enacted stigma experienced by the patients. Also, as in these low-resource settings, patients with mental disorders often remain undiagnosed, so any inadvertent inclusion of patients with minor mental health problems cannot be avoided. Additionally, the study did not assess other potential confounders such as substance use, personality traits, social support measures, or pre-existing subclinical depressive symptoms.

The high prevalence of depression and stigma throughout TB treatment has important clinical and public health implications. First, routine screening for depression should be integrated into TB care at all treatment stages, not just during the intensive phase. Second, targeted interventions to address both social and anticipated stigma are needed throughout the treatment period. Third, patient counseling should be strengthened as a core component of TB management, given its protective effect against stigma.

## Conclusion

Our study found high rates of depression and stigma among patients with TB in Bangladesh, with no significant differences between the intensive and continuation phases of treatment. These findings challenge the assumption that psychological burden necessarily decreases as treatment progresses and highlight the need for continuous mental health support throughout the TB treatment journey. Addressing depression and stigma should be integrated into comprehensive TB care to improve treatment outcomes, quality of life, and social reintegration of affected individuals.

## Declaration of competing interest

The authors have no competing interests to declare.

## Appendix Supplementary materials

Image, application 1

Image, application 2

## Footnotes

Supplementary material associated with this article can be found, in the online version, at doi:10.1016/j.ijregi.2025.100790.
