# Supplementary material for: Phase-wise comparison of depression and stigma among tuberculosis patients undergoing treatment in Dhaka, Bangladesh
Source: IJID Reg. 2025 Oct 18;17:100790. doi: 10.1016/j.ijregi.2025.100790 (PMC12662111; doi:10.1016/j.ijregi.2025.100790)
Supplement: Supplementary file 1 [file mmc1.docx]

**S1 Table. Distribution of responses to social stigma related items of the Van Rie's Stigma Scale**

|  | **Response Categories*** | | | |
| --- | --- | --- | --- | --- |
| **Items** | **Strongly disagree** | **Disagree** | **Agree** | **Strongly agree** |
| Some people may not want to eat or drink with friends who have TB | 176 (78.57) | 25 (11.16) | 22 (9.82) | 1 (0.45) |
| Some people feel uncomfortable about being near those with TB | 144 (64.29) | 7 (3.13) | 59 (26.34) | 14 (6.25) |
| If a person has TB, some community members will behave differently towards that person for the rest of his/ her life | 186 (83.04) | 23 (10.27) | 11 (4.91) | 4 (1.79) |
| Some people do not want those with TB playing with their children | 190 (84.82) | 20 (8.93) | 14 (6.25) | 0 (0.00) |
| Some people keep their distance from people with TB | 153 (68.30) | 17 (7.59) | 41 (18.30) | 13 (5.80) |
| Some people think that those with TB are disgusting | 176 (78.57) | 30 (13.39) | 14 (6.25) | 4 (1.79) |
| Some people do not want talk to others with TB | 178 (79.46) | 33 (14.73) | 10 (4.46) | 3 (1.34) |
| Some people are afraid of those with TB | 171 (76.34) | 32 (14.29) | 17 (7.59) | 4 (1.79) |
| Some people try not to touch others with TB | 166 (74.11) | 25 (11.16) | 27 (12.05) | 6 (2.68) |
| Some people may not want to eat or drink with relatives who have TB | 166 (74.11) | 28 (12.50) | 26 (11.61) | 4 (1.79) |
| Some people prefer not to have those with TB living in their community | 194 (86.61) | 16 (7.14) | 10 (4.46) | 4 (1.79) |

*Data was expressed as n (row %)

**S2 Table. Distribution of responses to anticipated stigma related items of the Van Rie's Stigma Scale**

|  | **Response Categories*** | | | |
| --- | --- | --- | --- | --- |
| **Items** | **Strongly disagree** | **Disagree** | **Agree** | **Strongly agree** |
| Some people who have TB feel hurt of how others react to knowing they have TB | 146 (65.18) | 32 (14.29) | 26 (11.61) | 20 (8.93) |
| Some people who have TB lose friends when they share with them they have TB | 165 (73.66) | 43 (19.20) | 13 (5.80) | 3 (1.34) |
| Some people who have TB feel alone | 145 (64.73) | 37 (16.52) | 35 (15.63) | 7 (3.13) |
| Some people who have TB keep their distance from others to avoid spreading TB germs | 100 (44.64) | 16 (7.14) | 40 (17.86) | 68 (30.36) |
| Some people who have TB are afraid to tell those outside their family that they have TB | 139 (62.05) | 31 (13.84) | 39 (17.41) | 15 (6.70) |
| Some people who have TB are afraid of going to TB clinics because other people may see them there | 168 (75.00) | 28 (12.50) | 26 (11.61) | 2 (0.89) |
| Some people who have TB are afraid to tell others that they have TB because others may think that they also have AIDS | 220 (98.21) | 3 (1.34) | 1 (0.45) | 0 (0.00) |
| Some people who have TB feel guilty because their family has the burden of caring for them | 175 (78.13) | 23 (10.27) | 22 (9.82) | 4 (1.79) |
| Some people who have TB will choose carefully who they tell about having TB | 102 (45.54) | 5 (2.23) | 40 (17.86) | 77 (34.38) |
| Some people who have TB feel guilty for getting because their smoking, drinking, or careless behavior | 197 (87.95) | 7 (3.13) | 1 (0.45) | 19 (8.48) |
| Some people who have TB are worried about having AIDS | 221 (98.66) | 2 (0.89) | 1 (0.45) | 0 (0.00) |
| Some people who have TB are afraid to tell their family that they have TB | 152 (67.86) | 15 (6.70) | 40 (17.86) | 17 (7.59) |

*Data was expressed as n (row %)

**S3 Table. Distribution of responses to PHQ-9 scale items**

|  | **Response Categories*** | | | |
| --- | --- | --- | --- | --- |
| **Characteristic** | **Not at all** | **Several days** | **More than half the days** | **Nearly everyday** |
| Little interest or pleasure in doing things | 119 (53.13) | 46 (20.54) | 36 (16.07) | 23 (10.27) |
| Feeling down, depressed or hopeless | 112 (50.00) | 42 (18.75) | 37 (16.52) | 33 (14.73) |
| Trouble falling or staying asleep, sleeping too much | 108 (48.21) | 43 (19.20) | 40 (17.86) | 33 (14.73) |
| Feeling tired or having little energy | 80 (35.71) | 33 (14.73) | 50 (22.32) | 61 (27.23) |
| Poor appetite or overeating | 124 (55.36) | 36 (16.07) | 33 (14.73) | 31 (13.84) |
| Feeling bad about yourself or that you are a failure or have let yourself or your family down | 162 (72.32) | 33 (14.73) | 20 (8.93) | 9 (4.02) |
| Trouble concentrating on things, such as reading the newspaper or watching the television | 146 (65.18) | 34 (15.18) | 30 (13.39) | 14 (6.25) |
| Moving or speaking so slowly that other people could have noticed or the opposite – being so fidgety or restless that you have been moving around a lot more than usual | 121 (54.02) | 40 (17.86) | 44 (19.64) | 19 (8.48) |
| Thoughts that you would be better off dead or of hurting yourself | 194 (86.61) | 18 (8.04) | 11 (4.91) | 1 (0.45) |

*Data was expressed as n (row %)
