# Supplementary material for: Phase-wise comparison of depression and stigma among tuberculosis patients undergoing treatment in Dhaka, Bangladesh
Source: IJID Reg. 2025 Oct 18;17:100790. doi: 10.1016/j.ijregi.2025.100790 (PMC12662111; doi:10.1016/j.ijregi.2025.100790)
Supplement: Supplementary file 2 [file mmc2.docx]

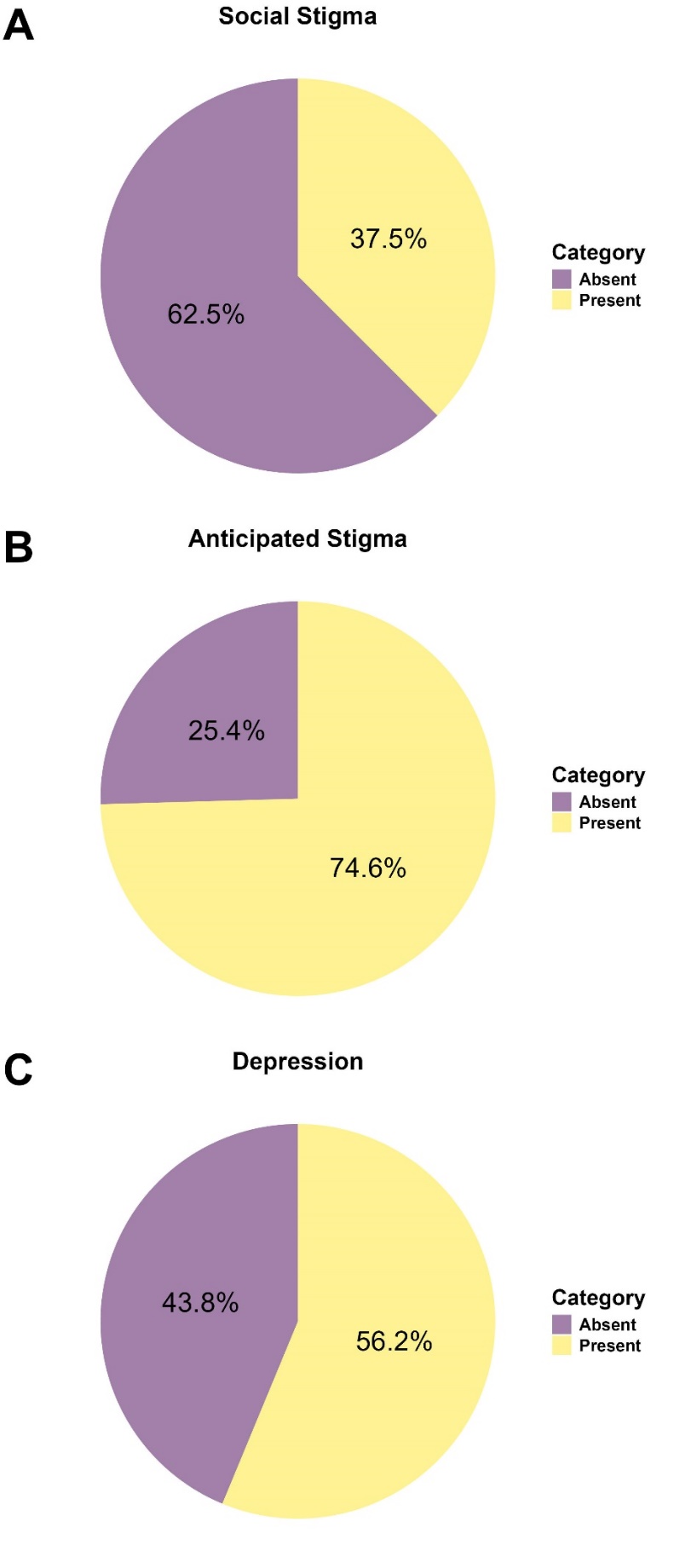


**Figure S1. Proportion of social stigma, anticipated stigma, and depression among the participants.**
